# Interleaved Array Transducer with Polarization Inversion Technique to Implement Ultrasound Tissue Harmonic Imaging

**DOI:** 10.3390/s20143915

**Published:** 2020-07-14

**Authors:** Chan Yuk Park, Jin Ho Sung, Eun Young Jeong, Hee Su Lee, Jong Seob Jeong

**Affiliations:** Department of Medical Biotechnology, Dongguk University, Seoul 04620, Korea; tanya00@naver.com (C.Y.P.); madeinjino@gmail.com (J.H.S.); chocoeun7@dongguk.edu (E.Y.J.) 22sooooo@dongguk.edu (H.S.L.)

**Keywords:** finite element analysis (FEA) simulation, interdigital bonding process, interleaved array transducer, polarization inversion technique, tissue harmonic imaging

## Abstract

In ultrasound tissue harmonic imaging (THI), it is preferred that the bandwidth of the array transducer covers at least the fundamental frequency f_0_ for transmission and the second harmonic frequency 2f_0_ for reception. However, it is challenging to develop an array transducer with a broad bandwidth due to the single resonance characteristics of piezoelectric materials. In this study, we present an improved interleaved array transducer suitable for THI and a dedicated transducer fabrication scheme. The proposed array transducer has a novel structure in which conventional elements exhibiting f_0_ resonant frequency and polarization-inverted elements exhibiting 2f_0_ resonant frequency are alternately located, and the thicknesses of all piezoelectric elements are identical. The performance of the proposed method was demonstrated by finite element analysis (FEA) simulations and experiments using a fabricated prototype array transducer. Using the proposed technique, f_0_ and 2f_0_ frequency ultrasounds can be efficiently transmitted and received, respectively, resulting in a 90% broad bandwidth feature of the transducer. Thus, the proposed technique can be one of the potential ways to implement high resolution THI.

## 1. Introduction

Recently, tissue harmonic imaging (THI) based on nonlinear phenomenon has begun to be widely used in echocardiography because it can provide a high resolution, improved signal-to-noise ratio (SNR), and reduced near-field artifacts [1,2,3,4,5,6,7,8,9]. In general, axial resolution can be improved by using higher frequencies and broader bandwidth signals. With higher frequencies of harmonic signals compared to the fundamental frequency, THI can improve axial resolution. Otherwise, a conventional THI transducer uses a narrower bandwidth to filter out frequencies from the fundamental beam, so the axial resolution can be reduced. Therefore, to increase the resolution of THI, the bandwidths for f_0_ transmission and 2f_0_ reception should be as broad as possible while minimizing overlap between the two components [10,11,12]. In order to increase the bandwidth of a transducer, a piezoelectric material with a high electromechanical coefficient can be used. A composite structure or optimized passive layers can contribute to broadening a bandwidth [13,14,15,16]. However, those methods have a limit to increase a bandwidth more than a certain level due to the single resonance feature of the piezoelectric material within the frequency range of interest, which is called an odd-order thickness-extensional vibration [17,18]. A capacitive micromachined ultrasound transducer (CMUT) and a piezoelectric micromachined ultrasound transducer (PMUT) can increase a bandwidth [19,20,21,22]. However, the inherent non-linear behavior of a CMUT is problematic for separating harmonic components from a target, and the CMUT requires a high bias voltage that results in safety issue [19,20]. The PMUT also has fabrication difficulty for high performance piezoelectric thin film and has a low electromechanical coupling coefficient [21,22].

In order to overcome this limitation, some researchers have proposed a method of alternately arranging f_0_ and 2f_0_ elements in a single array transducer known as an interleaved array transducer for THI [23,24,25]. This method can achieve the maximal bandwidths of the f_0_ and 2f_0_ elements, respectively, and physically separate received f_0_ and 2f_0_ frequency components without using a band pass filter. However, the fabrication process of the conventional interleaved array transducer is complicated because the thicknesses of two types of piezoelectric elements are different. That is, the bonding process of a matching layer, a backing layer, and a flexible printed circuit board (FPCB) to the piezoelectric layer with a different thickness is quite challenging. 

In this study, we propose a novel interleaved array transducer for THI that is capable of overcoming the aforementioned issue. The proposed transducer also consists of f_0_ and 2f_0_ elements, which are placed alternately, but the thickness of the 2f_0_ piezoelectric element is the same as that of the f_0_ piezoelectric element. To achieve this effect, the polarization inversion technique (PIT), which is capable of generating an even-order thickness-extensional vibration, was employed for the 2f_0_ element [26,27,28,29]. The PIT can increase the center frequency of the transducer by combining multiple piezoelectric layers with opposite poling directions. In this structure, the center frequency can be increased even though the total thickness of the piezoelectric layer is not thinner [28,29]. Since the thicknesses of all elements are identical, the fabrication process for interleaving elements is more convenient compared to the conventional fabrication method. Unlike the conventional PIT, which mainly uses LiNbO_3_ material and a co-firing fabrication technique, in the proposed technique, a common PZT-5H was selected as the piezoelectric material, and a mechanical bonding technique using an unloaded epoxy was used for PIT implementation. The performance of the proposed array transducer was evaluated by finite element analysis (FEA) simulation, and a prototype array transducer was subsequently fabricated for experimental demonstration. 

The fundamental operational principle, design specifications, and FEA simulation results of the proposed array transducer are described in the Section 2. Experimental validation using the prototype array transducer and method of transducer fabrication are described in the Section 3. The discussion and conclusion are drawn in Section 4.

## 2. Materials and Methods

### 2.1. Polarization Inversion Technique

**Figure 1:**
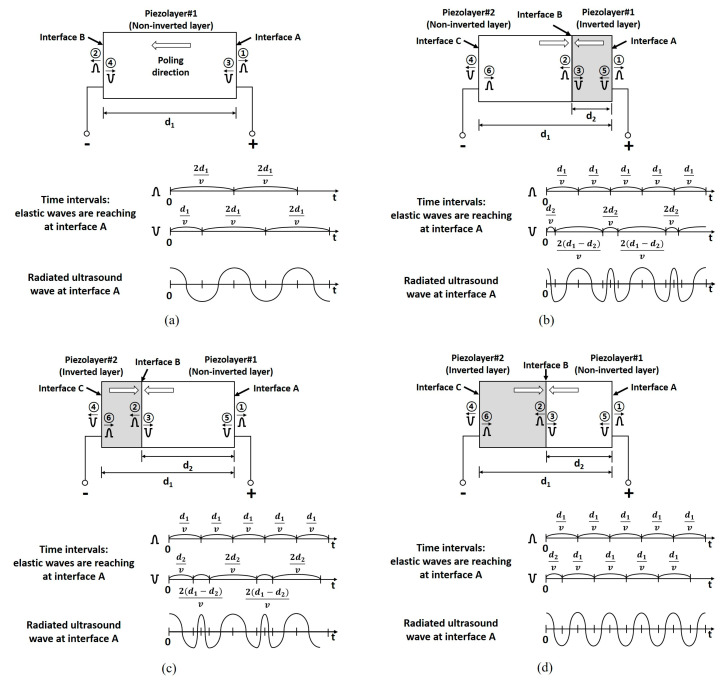
Operational principle of the conventional and polarization inversion technique (PIT) models: (**a**) Conventional structure with a single piezoelectric layer, (**b**) polarization-inverted layer structure with a 0.25 inversion ratio (front-side inversion model), (**c**) polarization-inverted layer structure with a 0.25 inversion ratio (back-side inversion model), and (**d**) the PIT with a 0.5 inversion ratio (half-inversion model). Note that the time and frequency responses of the radiated ultrasound waves at interface A for each case were also demonstrated by computational simulation, as shown in Figure 2.

**Figure 2:**
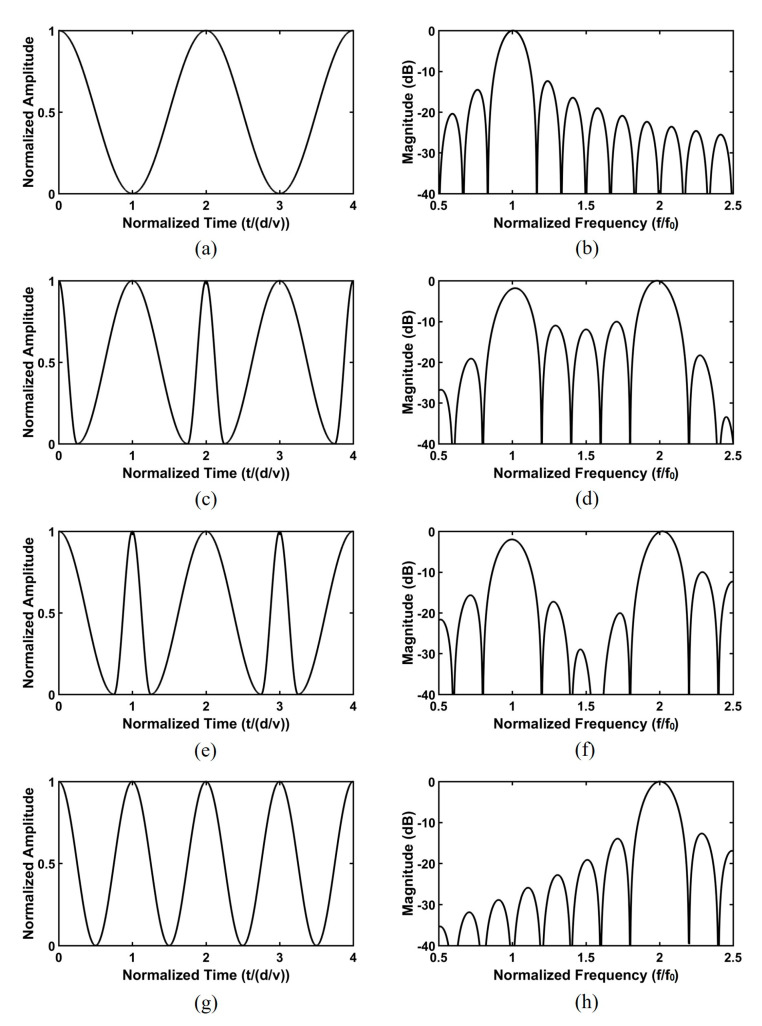
Time and frequency responses of the radiated ultrasound waves at interface A for each case in Figure 1 based on MATLAB simulations: (**a**) Time and (**b**) frequency domain responses of the conventional model, (**c**) time and (**d**) frequency domain responses of the PIT with a 0.25 inversion ratio (front-side inversion model), (**e**) time and (**f**) frequency domain responses of the PIT with a 0.25 inversion ratio (back-side inversion model), and (**g**) time domain and (**h**) frequency domain responses of the PIT with a 0.5 inversion ratio (half inversion model).

In the PIT, two sub-piezoelectric layers are bonded with opposite poling directions, and they operate as a single-piezoelectric layer. The PIT can simultaneously generate f_0_ and 2f_0_ based on odd-order and even-order thickness-extensional vibrations [26,29]. Only two electrodes are formed at the top and bottom sides of the combined piezoelectric layer. The position of the resonance frequencies and electrical impedances can be controlled depending on the thickness ratio of the two bonded layers [26,28,29]. The operational principle can be explained by elastic wave propagation, as shown in Figure 1. Figure 1a shows a single piezoelectric layer with one poling direction. When a voltage is applied to the piezoelectric layer in the same direction as the poling direction, the elastic waves with positive-polarity are propagated outward from interface A and B. The phases of the reflected signals at interface A and C are inverted due to the difference of the acoustic impedance between the active layer and medium. Thus, the elastic waves with negative-polarity are generated and propagated in the opposite direction based on principles of action and reaction. When observing the wave arrival time at interface A, the positive- and negative-polarity waves reach a 2d_1_/v interval, and, thus, an ultrasound wave with the fundamental resonant frequency can be generated. In the case of Figure 1b with the PIT, two piezoelectric layers were combined together with opposite poling directions. In this scheme, the inversion ratio defined by the thickness of the inverted piezoelectric layer to the thickness of the entire piezoelectric layer was 0.25. In the PIT, the thinner layer is called the inverted layer, and when the inverted layer is located at the front side of the transducer, we call it a front-side inversion model. When the voltage is applied, the positive-polarity waves reach interface A with the d_1_/v interval. The negative-polarity waves reach interface A with 2d_2_/v and 2(d_1_-d_2_)/v intervals alternatively. When the position of the inverted layer is changed to back side of the transducer, as shown in Figure 2c (which is called a back-side inversion model), the operational principle is the same as in Figure 2b except for the staring time interval. In the case of Figure 1d with the PIT, two piezoelectric layers with a 0.5 inversion ratio were combined together for what is called a half-inversion model. When the voltage is applied, the positive- and negative-polarity waves reach interface A with a d_1_/v interval, which is half the time interval of Figure 1a. Thus, when the inversion ratio became 0.5, the resonance frequency could be increased to 2f_0_. Note that the total thickness of the piezoelectric layer with a 0.5 inversion ratio could be identical to that of a conventional piezoelectric layer with a fundamental resonance frequency.

In order to more clearly verify the time and frequency responses of the radiated ultrasound waves at interface A for each case in Figure 1, computational simulation was conducted by using MATLAB (The MathWorks, Inc., Natick, MA, USA). In this simulation, the center frequency was assumed to be 5 MHz, which was normalized in the final step. The propagation velocity was assumed to be 4192 m/s considering the PZT-5H material. In addition, for front-side inversion, back-side inversion, and half-inversion models, d_1_ = 1, d_2_ = 0.25, 0.75, and 0.5, respectively. The fundamental frequency f_0_ of Figure 1a was generated as shown in Figure 2a,b. In the case of the PIT with a 0.25 inversion ratio in Figure 1b,c, the f_0_ and 2f_0_ peak frequencies were generated (Figure 2c–f). The performance difference between the two models were the level of the center valley. The front-side inversion model provided a relatively shallow depth of the valley compared to that of the back-side inversion model. Thus, the front-side inversion model can be used for a broad bandwidth transducer, while the back-side inversion model can be used for a dual-peak transducer [29,30].

When the inversion ratio was 0.5, like in Figure 1d, the center frequency of the PIT was 2f_0_, even though the thickness of the PIT model was the same as that of the conventional model described in Figure 2g,h. Unlike the previous interleaved array transducer, which used piezoelectric elements of different thicknesses to generate f_0_ and 2f_0_ ultrasounds, respectively, all piezoelectric elements of the proposed transducer have the same thickness. Therefore, the array transducer for THI can be efficiently fabricated including the bonding process for a matching layer, a backing layer, and an FPCB. In addition, the proposed transducer used a conventional mechanical bonding process and common piezoelectric material (PZT-5H), which are widely used in array transducers. Note that most previous studies about the PIT were conducted for a single element transducer using LiNbO_3_ and complicated co-firing methods [17,18].

### 2.2. Design of Interleaved Array Transducer Based on FEA Simulation

For the performance evaluation of the proposed array transducer, an FEA simulation using the PZFlex software (OnScale, Cupertino, CA, USA) was conducted. The center frequencies for transmission and reception were chosen as 5 and 10 MHz, respectively, based on a commercial linear array transducer for THI. A common PZT-5H (CTS3203HD, CTS Corporation, Lisle, IL, USA) was used as the piezoelectric material, and a 2-2 composite structure based on the sub-dicing process was employed for a more efficient aspect ratio (element width/element height) less than 0.5 [31,32,33,34,35]. The inversion ratio was chosen as 0.5 to generate only 2f_0_ frequency components. The piezoelectric element width was 301 μm, and the kerf width was 38 μm. After sub-dicing, the piezoelectric element width was 75 μm. The total thickness of the piezoelectric layer was 280 μm, and the aspect ratio of the sub-elements was thus 0.27. Two matching layers, i.e., aluminum powder (Metalplayer, Incheon, Korea) filled epoxy for the 1^st^ matching layer and EPO-TEK 301 (Epoxy Technology, Billerica, MA, USA) for the 2^nd^ matching layer were added on the piezoelectric layer, and tungsten powder (TaeguTec, Deagu, Korea) filled epoxy was used for a backing layer. The reference frequency used to design two matching layers was 1.5f_0,_ which is middle value between f_0_ and 2f_0_. The FPCB and bonding layer were included in this simulation, and the medium was water. Since the goal of this study was the performance demonstration of the proposed method, only eight elements were fabricated, i.e., four f_0_ and four 2f_0_ elements. Table 1 describes the design specifications of the proposed transducer, and Table 2 shows the material properties of the PZT-5H used in FEA simulation.

### 2.3. FEA Simulation Results

**Figure 3:**
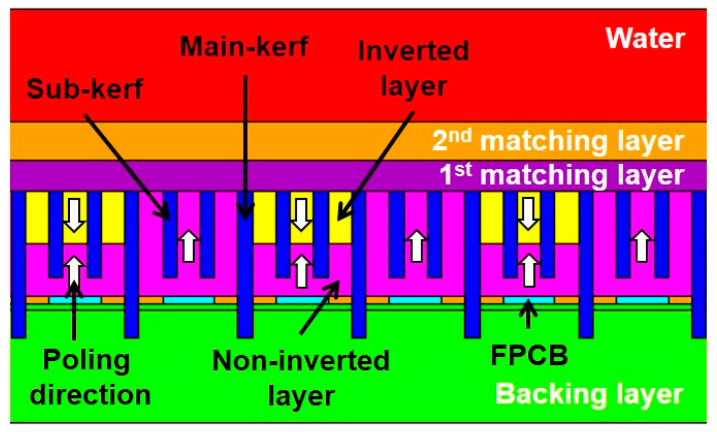
FEA-based schematic diagram of the proposed array transducer using the PIT for tissue harmonic imaging (THI). The array transducer was composed of the conventional elements for transmitting f_0_ ultrasounds and the PIT elements for receiving reflected 2f_0_ ultrasounds. The conventional elements exhibited the same poling direction, but the PIT elements exhibited the opposite poling direction. The identical matching layers were applied to the proposed model.

**Figure 4:**
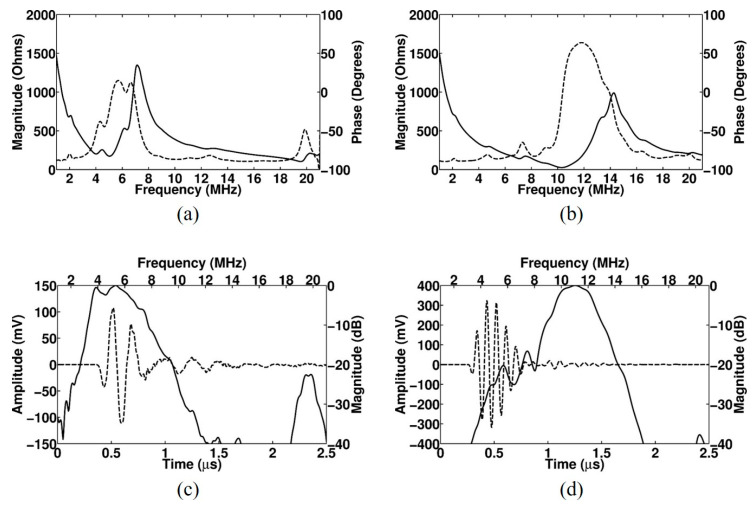
FEA simulated electrical impedance (solid line: magnitude; dashed line: phase) and pulse–echo response (solid line: frequency-domain spectrum; dashed line: time-domain waveform) of the proposed linear array transducer with matching layers: (**a**) Electrical impedance of the conventional element, (**b**) electrical impedance of the PIT element, (**c**) pulse–echo response of the conventional element, and (**d**) pulse–echo response of the PIT element.

Figure 3 shows a cross-sectional image of the proposed array transducer obtained from FEA-based software (OnScale, Cupertino, CA, USA). Only six elements are shown from the simulated eight elements. The conventional elements and PIT elements with a 0.5 inversion ratio were alternatively arranged, and identical matching layers were applied to all elements. The thicknesses of the piezoelectric layers for f_0_ ultrasound transmission and reflected 2f_0_ ultrasound reception were identical to each other even though the resonance frequencies were different. Figure 4 shows the electrical impedance and pulse–echo response of the conventional element and the PIT element. In the case of the conventional element, the electrical impedance was 173 Ω at a resonance frequency of 5 MHz (Figure 4a), and the PIT element was 26.2 Ω at a resonance frequency of 10.3 MHz (Figure 4b). In the case of the pulse–echo simulation, the conventional element had a 5.3 MHz center frequency with a −6 dB bandwidth of 71.1% (Figure 4c). On the other hand, the PIT element had a 11 MHz center frequency with a −6 dB bandwidth of 28.5% (Figure 4d). From those results, it was verified that the PIT elements only generated 2f_0_ resonance frequency components successfully.

## 3. Experimental Validation 

### 3.1. Fabrication of Prototype Array Transducer with PIT and Novel Interleaved Technique

**Figure 5:**
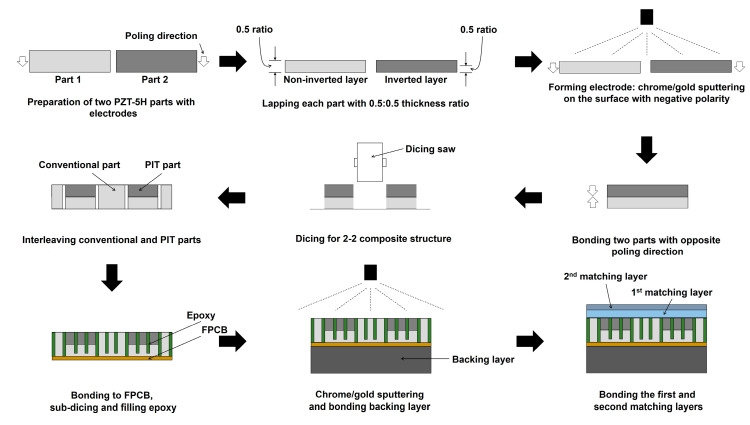
Fabrication process of the proposed prototype array transducer. The improved interleaved technique was employed for this fabrication.

In order to experimentally demonstrate the performance the proposed transducer, a prototype array transducer with the PIT was built based on the FEA-simulated design specifications. First, three bulk-type PZT-5H plates with dimensions of 4 × 15 × 0.5 mm were prepared, as described in Figure 5. One of them was used for the conventional elements, and two of them were used for the PIT elements. One part was lapped to 280 μm for the 5 MHz resonance. For the PIT elements, two parts with negative polarity were lapped as 140 μm. Chrome/Gold (500 Å/2000 Å) was sputtered on the lapped surface of each part to form the electrodes. After bonding two parts with opposite poling directions by using epoxy (EPO-TEK 353ND, Epoxy Technology, Billerica, MA, USA), they were diced to form a 2-2 composite using a dicing saw (DAD322, DISCO Corp., Tokyo, Japan). After dicing the conventional elements with the same process, two types of piezoelectric components were alternatively assembled and bonded to the FPCB. After sub-dicing, unloaded epoxy was used to fill the kerfs. Subsequently, they were bonded to the backing layer, which was made using the mixture of tungsten powder and EPO-TEK 301. The first matching layer with a thickness of 80 μm and the second matching layer with a thickness of 100 μm were bonded on the surface of piezoelectric material in sequence. In the case of the first matching layer, aluminum powder with a 1–3 μm particle size and EPO-TEK 301 epoxy were mixed, resulting in a 70% volume fraction ratio. The reference frequency used to make two matching layers was 1.5f_0,_ which was a middle value between f_0_ and 2f_0_.

**Figure 6:**
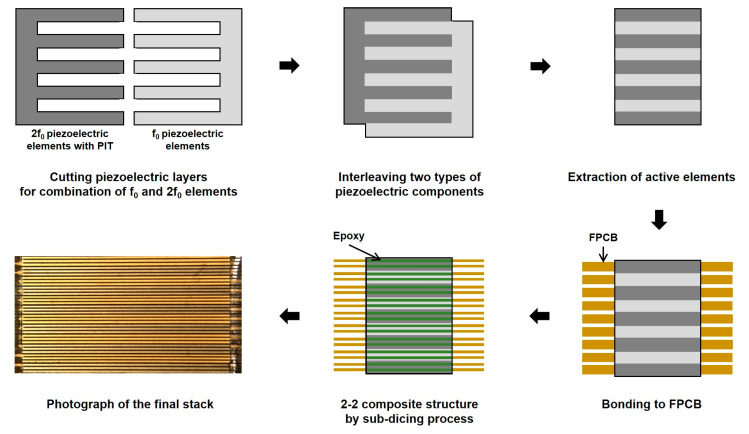
Fabrication process of the proposed prototype array transducer focusing on the novel interleaved technique.

**Figure 7:**
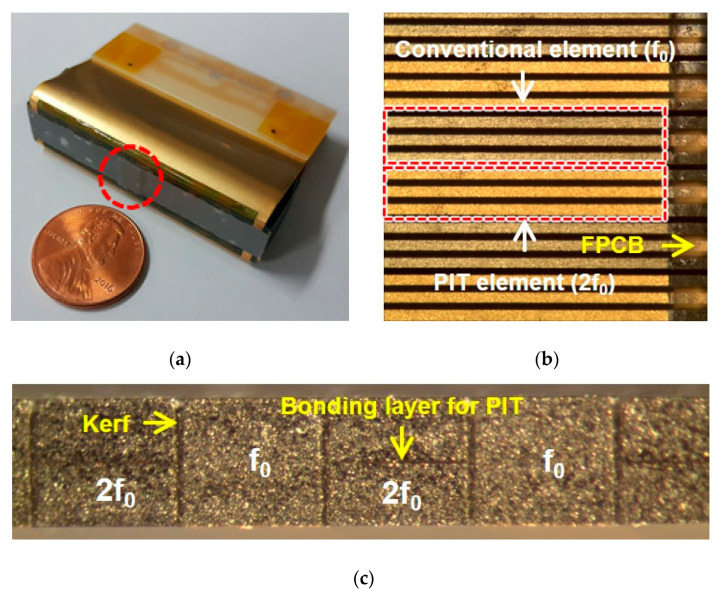
Photographs of the prototype array transducer: (**a**) Prototype array transducer after bonding matching and backing layers, (**b**) magnified photograph of a red-dotted circle in (**a**) before bonding matching layers (top view), and (**c**) magnified photograph of (**b**) before the sub-dicing process (cross sectional side view).

Figure 6 shows the manufacturing process of the prototype array transducer, focusing on the process of alternately assembling arrays, i.e., the interleaving process. In the case of the conventional interleaving process, the interdigital bonding process, two types of elements are combined up and down [36,37,38,39,40]. Therefore, the interleaving process should be performed while looking at the side, which makes the work very inconvenient and parts easily damageable. However, the proposed interleaving method is more efficient than conventional techniques because it can monitor and assemble the two types of elements while looking down. Therefore, it is possible to more safely and accurately adjust the kerf between elements. Figure 7a shows the fabricated prototype array transducer after bonding matching and backing layers. A red-dotted circle indicates the position of the eight elements. Figure 7b shows a magnified photograph of the red-dotted circle in Figure 7a, which was composed of the conventional element and the PIT element. The alternately arranged f_0_ and 2f_0_ elements are clearly shown in Figure 7b. The bonding layer of the PIT can be seen in Figure 7c, and the same thickness of the two types of elements can be confirmed.

### 3.2. Experimental Results

**Figure 8:**
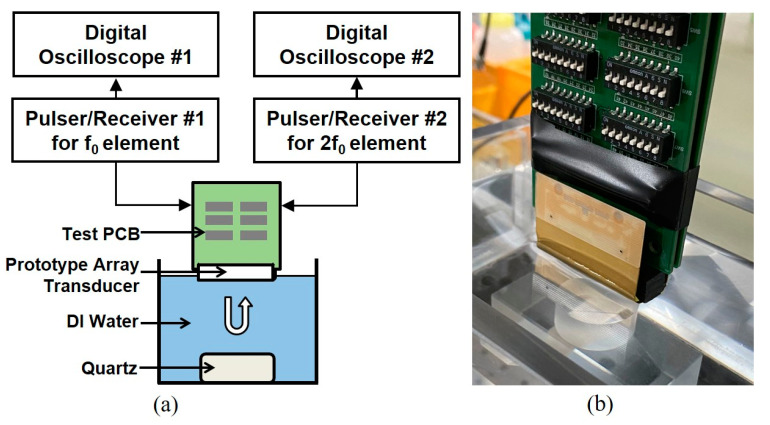
Experimental setup for pulse–echo measurement. Two pulsers/receivers were used for independent pulse–echo test of each element and the test for transmitting f_0_ and receiving reflected 2f_0_ frequency components: (**a**) Schematic diagram and (**b**) photograph of (**a**).

**Figure 9:**
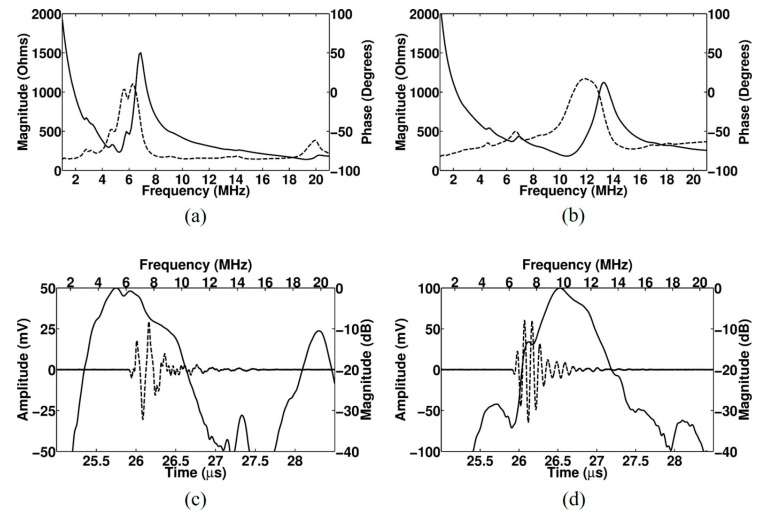
Measured electrical impedance (solid line: magnitude; dashed line: phase) and pulse–echo responses (solid line: frequency-domain spectrum; dashed line: time-domain waveform) of the proposed linear array transducer with matching layers: (**a**) Electrical impedance of the conventional element, (**b**) electrical impedance of the PIT element, (**c**) pulse–echo response of the conventional element, and (**d**) pulse–echo response of the PIT element.

**Figure 10:**
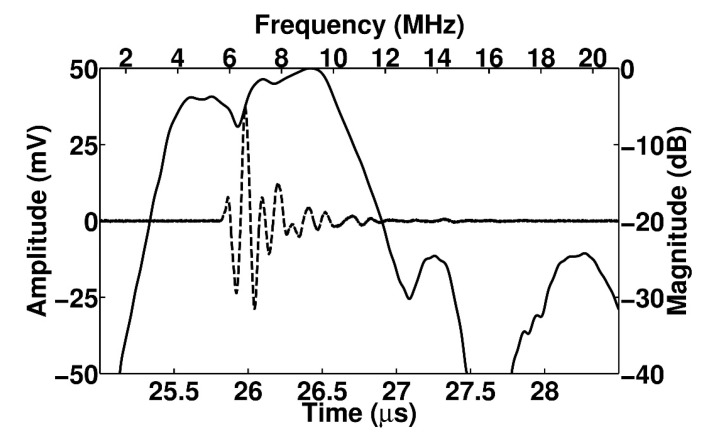
Measured pulse–echo response of the proposed linear array transducer when the f_0_ element Table 2. f_0_ element receives the reflected ultrasounds: (solid line: frequency-domain spectrum; dashed line: time-domain waveform).

The electrical impedance and pulse echo response were measured by using an impedance analyzer (4294 A, Keysight, San Jose, CA, USA) and a pulser/receiver (5073PR, Olympus, WA, USA), respectively. Figure 8 shows the experimental setup to measure the pulse echo response. Figure 9 shows the measured electrical impedance and pulse–echo response using the fabricated prototype array transducer. The electrical impedance was measured in the air medium. The electrical impedance of the conventional f_0_ element and 2f_0_ element with the PIT were 234.8 Ω at a resonance frequency of 5.2 MHz and 183 Ω at a resonance frequency of 10.5 MHz, respectively. The measured center frequency of the f_0_ element was 5.8 MHz with a −6 dB bandwidth of 55%. The 2f_0_ element had a 10.2 MHz center frequency with a −6 dB bandwidth of 31.2%. Thus, it was verified that the conventional elements can transmit f_0_ ultrasounds and the PIT elements can receive reflected 2f_0_ ultrasounds even though the total thicknesses of the two types of elements were identical. Thus, THI can be implemented by using the proposed transducer. Figure 10 shows the pulse–echo response when the f_0_ element transmitted the impulse signal, and the 2f_0_ element received the reflected ultrasounds. From this experiment, it can be considered that the total −6 dB bandwidth of the proposed transducer was 90%, including a shallow valley point. Considering that the typical −6 dB bandwidth of the PZT-5H was about 70%, the performance of the proposed method was about 20% higher than the conventional model.

## 4. Discussion and Conclusions

In order to efficiently implement THI, the bandwidth of the ultrasound array transducer should be broad enough to cover from f_0_ to 2f_0_. However, since most single-layer piezoelectric materials have an odd-order resonance and a single resonance is normally dominant within the region of interest frequency range, it is challenging to make a broad bandwidth array transducer. The interleaved array transducer does not require a broad bandwidth because it physically separates the transmitting and receiving elements without using the band pass filter, and each full frequency bandwidth for f_0_ and 2f_0_ can be used for THI. One of the main limitations of this method is that the thicknesses of two types of elements are different and thus, the fabrication process is difficult. In this study, we proposed an improved interleaved array transducer exhibiting the uniform thickness of the piezoelectric layer even though two types of elements with different resonance frequencies are mixed alternatively. This can be achieved by employing the PIT, especially with an 0.5 inversion ratio capable of generating a 2f_0_ frequency component based on the conventional thickness for the f_0_ resonance frequency.

In the FEA simulation and experiment using a prototype array transducer, f_0_ and 2f_0_ frequency components were successfully generated. To assemble pre-diced two types of elements, two plates should be carefully interleaved. Different from the conventional interleaved technique, we proposed a modified fabrication process by combining two plates side by side. In the conventional method, it is difficult to align the piezoelectric rods because most of the process should be performed by looking at the side, but the proposed method is performed while looking at the top, so relatively convenient and accurate alignment can be achieved. That is, since the thicknesses of the two plates are the same, it is easy to align the elements and to combine the FPCB and the backing layer. 

To increase the bandwidth of each element, a matching layer should be attached. Since the center frequency of this array transducer is different, the center frequency used for designing the matching layer should be optimized. Normally, the thickness of the matching layer is a quarter wavelength in the matching material. That can be controlled depending on the application. In our design, the reference frequency was 1.5 f_0_ considering the f_0_ and 2f_0_ center frequencies of two types of elements. Thus, there will be reduction of resolution for f_0_ and 2f_0_ frequency components compared to the conventional interleaved array transducer. In the case of the experiment of transmitting f_0_ ultrasounds and receiving reflected ultrasounds with the 2f_0_ element, the total −6 dB bandwidth was 90%, which is a broad bandwidth compared to the conventional PZT-5H with a bandwidth of about 70%. That is the proposed method that can implement a broad bandwidth by using two types of independent elements rather than a single element transducer. 

In the proposed technique, a 1.5 f_0_ frequency was used for designing two matching layers, and thus, the −6 dB bandwidths of the conventional elements and the PIT elements may be reduced compared to the best condition of two types of elements. Therefore, the imaging performance of the proposed technique may be slightly reduced compared to the conventional interleaved array transducer. However, if more optimized reference frequency and thicknesses of matching layers are chosen, the −6 dB bandwidths of the conventional elements and the PIT elements can be increased more. Like the conventional interleaved array transducer, the proposed method also has a grating lobe issue due to its large pitch. In order to minimize grating lobe generation, controlling the amplitude of the input signal or using a variable pitch will help to solve this problem. The proposed technique has advantages about several manufacturing processes compared to other interleaved transducers for THI, but it requires manufacturing challenges and high costs compared to conventional non-interleaved THI transducers. The interleaved array transducer is not a replacement for conventional THI transducers, but it is one of the useful transducers to implement THI. Each of them has advantages and disadvantages. This manuscript provides information on an improved interleaved array transducer that can also be a useful THI transducer. Additionally, since the imaging system has not yet been prepared, pulse–echo measurements were mainly performed. In the future, we plan to conduct tissue harmonic B-mode (brightness mode) imaging tests using the imaging system.

In this study, we demonstrated that the PIT can be applied to an ultrasound linear array transducer for THI. The proposed transducer is composed of single layer piezoelectric elements for transmitting f_0_ ultrasounds and PIT elements for receiving 2f_0_ ultrasounds, and these are alternately arranged. In the proposed transducer, each element has a unique resonant frequency, so not all elements need to have a broad bandwidth for THI. If the design specifications of the proposed transducer are more optimized, the frequency bandwidth of f_0_ and 2f_0_ can be further increased. Unlike previous studies that used piezoelectric elements of different thicknesses for f_0_ and 2f_0_ ultrasounds, all piezoelectric elements of the proposed transducer have the same thickness, which makes it possible to efficiently manufacture array transducers for THI. To make an interleaved type transducer, a novel interleaved method was introduced, and its performance was also successfully demonstrated. Unlike the conventional method, it is possible to arrange elements while looking down from the top, so more convenient operation is possible. Additionally, the proposed transducer used a conventional physical bonding process to form the polarization-inverted piezoelectric layer, and a popular piezoelectric material, i.e., PZT-5H, was employed. Our simulation and experimental results also showed that multiple piezoelectric layers with a 0.5 inversion ratio can generate higher frequency ultrasounds. Therefore, an interleaved array transducer based on the PIT could be a promising way to conduct high resolution THI.

## Figures and Tables

**Table 1:** Design specifications of the proposed transducer used in the finite element analysis (FEA) simulation.

	Conventional f_0_ Element	PIT 2f_0_ Element
Center frequency [MHz]	5	10
Element number	4	4
Element width [μm]	301	
Sub-element width [μm]	75	
Kerf size [μm]	38	
Total thickness of piezoelectric layer [μm]	280	
Thickness of 1st matching layer [μm]	80	
Thickness of 2nd matching layer [μm]	100	
Piezoelectric material	PZT-5H	
1st Matching layer material	Aluminum powder filled epoxy	
2nd Matching layer material	Unloaded epoxy	
Backing layer material	Tungsten powder filled epoxy	

**Table 2:** Material properties of the PZT-5H used in the FEA simulation.

PZT-5H (CTS3203HD)		
Elastic stiffness $c^{E}\;\left(10^{10}\;N/m^{2}\right)$	$c_{11}^{E}$	13.7
	$c_{12}^{E}$	8.8
	$c_{13}^{E}$	9.23
	$c_{33}^{E}$	12.6
	$c_{44}^{E}$	2.2
	$c_{66}^{E}$	2.5
Mechanical Q		90
Dielectric permittivity $\epsilon^{s}/\epsilon_{0}$	$\epsilon_{11}^{s}$	1305.8
	$\epsilon_{33}^{s}$	1200.2
Piezoelectric stress e $\left(C/m^{2}\right)$	$e_{13}$	−9.4
	$e_{33}$	22.5
	$e_{15}$	16.1
Density $\left(\mathrm{kg}/m^{3}\right)$	ρ	7820
